# Glycosylphosphatidylinositol-Anchored Proteins in *Fusarium graminearum*: Inventory, Variability, and Virulence

**DOI:** 10.1371/journal.pone.0081603

**Published:** 2013-11-29

**Authors:** William R. Rittenour, Steven D. Harris

**Affiliations:** 1 Department of Plant Pathology and Center for Plant Science Innovation, University of Nebraska Lincoln, Lincoln, Nebraska, United States of America; 2 Chestnut Brew Works, LLC, Morgantown, West Virginia, United States of America; University of Wisconsin - Madison, United States of America

## Abstract

The contribution of cell surface proteins to plant pathogenicity of fungi is not well understood. As such, the objective of this study was to investigate the functions and importance of glycosylphosphatidylinositol-anchored proteins (GPI-APs) in the wheat pathogen *F. graminearum*. GPI-APs are surface proteins that are attached to either the membrane or cell wall. In order to simultaneously disrupt several GPI-APs, a phosphoethanolamine transferase-encoding gene *gpi7* was deleted and the resultant mutant characterized in terms of growth, development, and virulence. The Δ*gpi7* mutants exhibited slower radial growth rates and aberrantly shaped macroconidia. Furthermore, virulence tests and microscopic analyses indicated that Gpi7 is required for ramification of the fungus throughout the rachis of wheat heads. In parallel, bioinformatics tools were utilized to predict and inventory GPI-APs within the proteome of *F. graminearum*. Two of the genes identified in this screen (FGSG_01588 and FGSG_08844) displayed isolate-specific length variability as observed for other fungal cell wall adhesion genes. Nevertheless, deletion of these genes failed to reveal obvious defects in growth, development, or virulence. This research demonstrates the global importance of GPI-APs to *in planta* proliferation in *F. graminearum*, and also highlights the potential of individual GPI-APs as diagnostic markers.

## Introduction

 Although several genes have been implicated in the virulence of plant pathogenic fungi, few have been found to encode cell surface proteins. In particular, very little is known about the role of proteins that are covalently attached to the carbohydrate backbone of the fungal cell wall. Such proteins are often termed “mannoproteins” due to their high glycosylation level, and they typically constitute a significant portion of fungal cell walls. For example, the cell wall of *F. graminearum* is calculated to contain ~4.5% protein by weight [1]. These mannoproteins form an electron-dense layer on the periphery of an electron-light layer representing the carbohydrate backbone [2,3]. Confirmed functions of these proteins include adhesion and modification of cell wall carbohydrates [4,5], though several additional roles have been predicted based on similarity to proteins of known function (see below).

In *Saccharomyces cerevisiae*, two types of covalently attached cell wall proteins have been described. The first class is the PIR (proteins with internal repeats) proteins. Much less is known about this class of cell wall protein compared to their glycosylphosphatidylinositol-anchored proteins (GPI-APs) counterparts (see below), though an alkali sensitive bond mediates their attachment to the cell wall. To date, PIR proteins with known functions include β1,3 glucanases and heat shock proteins [2]. The second (and more widely studied) class of fungal cell wall proteins is the GPI-APs. Outside of fungi, this class mostly consists of membrane-localized proteins, but in fungi, many are instead released and covalently attached to the cell wall [6]. GPI-APs typically contain conserved regions that allow for their prediction based on primary sequence analysis, including an N-terminal signal peptide and C-terminus anchor addition signal [7]. As such, several genomes have been mined for predicted GPI-APs, and a general trend has been the identification of adhesins, carbohydrate-modifying enzymes, aspartyl proteases, and proteins of unknown function [7-10]. Notably, several of the genes that encode GPI-APs (particularly the adhesins) contain internal sequence repeats that vary among different strains of the same species [11-13] (note that these repeats are different from the “internal repeats” of the PIR proteins, which contain a conserved internal repeat sequence that is necessary for attachment to the cell wall [14]). Although proteomes of model fungi and some human pathogens have been mined for GPI-APs [11,13], the proteomes of plant pathogenic fungi have yet to be investigated. 

GPI-APs undergo several processing events before becoming incorporated into the plasma membrane or cell wall. First, the GPI-anchor is sequentially “built” from a phosphatidylinositol (PI) molecule. To this PI is first added a glucosamine followed by three sequential mannose groups. The early steps of GPI construction occur on the cytoplasmic face of endoplasmic reticulum (ER) before a flippase transfers the head group to the lumenal face [15,16]. The N-terminal signal peptide of the pro-GPI-AP targets it to the endoplasmic reticulum, where the C-terminal anchor addition sequence is recognized and further processed by the GPI-transamidase complex [17]. The pro-GPI-AP is added to the amino group of a phosphoethanolamine (PEA) attached to the third mannose residue. In addition to this terminal PEA group, there is a PEA group attached to each of the three mannose residues in the GPI-anchor. Although their precise function is unclear, these PEA groups contribute significantly to the function of GPI-APs, as mutations in PEA-transferase genes *mcd4* (PEA attachment to first mannose residue) and *gpi13* (third mannose residue) cause drastic hyphal phenotypes in *Neurospora crassa* [18]. In *S. cerevisiae* and *Candida albicans*, the PEA on the second mannose residue likely contributes to covalent attachment of cell wall GPI-APs, as deletion of PEA transferase Gpi7 causes mislocalization of cell wall-localized, but not membrane-localized, GPI-APs [19]. Although the molecular components of GPI-AP construction complexes (i.e. transamidase, PEA transferase complexes) are beginning to be elucidated in filamentous fungi [18,20], a viable *gpi7* deletion mutant has yet to be generated.

Cell wall proteins likely contribute significantly to the fitness of plant pathogenic fungi. Given that many predicted GPI-APs encode proteins with enzymatic functions (i.e. aspartyl proteases), these enzymes may contribute to the degradation of host tissues. Indeed, GPI-anchored aspartyl proteases and secreted lysophospholipases contribute to the virulence of several human pathogenic fungi [21-24]. Also, cell wall proteins may contribute to the structural integrity of the cell wall itself, thereby protecting the pathogen from environmental stresses encountered within host tissue [e.g. host pathogenesis-related (PR) proteins]. Accordingly, overexpression of *pir2*, which encodes a *S. cerevisiae* cell wall protein, in the tomato wilt pathogen *F. oxysporum* increases its resistance to the tomato PR protein osmotin [25]. Also, the predicted GPI-anchored extracellular matrix protein Emp1 in the rice blast fungus *Magnaporthe oryzae* is necessary for proper appressorium formation and function, likely because it enables *M. oryzae* to withstand the high turgor pressure necessary for host penetration [26]. Given the proposed roles for cell wall proteins in fungal morphogenesis and pathogenicity and the lack of information regarding the presence and function of these proteins in plant pathogenic fungi, the objectives of this study were to investigate the roles of GPI-APs during *F. graminearum* infection of wheat by characterizing a Gpi7 homologue as well as several predicted GPI-APs identified using a bioinformatic approach.

## Materials and Methods

### Strains and culture conditions

Proteomic analysis and gene deletions were performed using strain PH-1 (NRRL 31084). Field isolates of *F. graminearum* collected from wheat heads in Nebraska were kindly provided by Julie Breathnach, Christy Jochum, and Dr. Gary Yuen, from the Dept. of Plant Pathology, University of Nebraska-Lincoln. No specific permissions were required for these isolates as they were obtained through routine disease surveys that did not involve endangered or protected species. All strains were maintained as mycelial suspensions in 30% glycerol at -80°C. Strains were grown on V8 agar [27] and conidia were collected from YMA plates in sterile distilled water and filtered through miracloth (Calbiochem). Conidiophores were imaged after removal from the surface of YMA plates incubated at 28°C for four days. Conidiation was assessed by inoculating 100 ml of CMC [28] with 5 μl of 1 X 10^5^ macroconidia. CMC cultures were incubated at 200 RPM at room temperature for 5 days, and concentrations of macroconidia were measured using a hemacytometer. Dimensions of macroconidia were measured using differential interference contrast microscopy and IPLab Imaging Software (Scanalytics, Inc). Biomass was assessed by inoculating 50 ml liquid YMA with 5 μl of 1 X 10^5^ macroconidial suspension, followed by incubation on a rotorary shaker set at 28°C and 200 RPM for 3 days. The resulting mycelium was vacuum filtered and further dried at 60°C for 16 hours to obtain the dry biomass. Sexual crosses were performed on carrot agar as previously described [27,29]. To assess cell wall defects, strains were tested for growth on YMA containing calcofluor white (fluorescent brightener 28; Sigma) and Congo red (Sigma) as described previously [30]. 

### Global disruption of GPI protein anchoring

**Figure 1:**
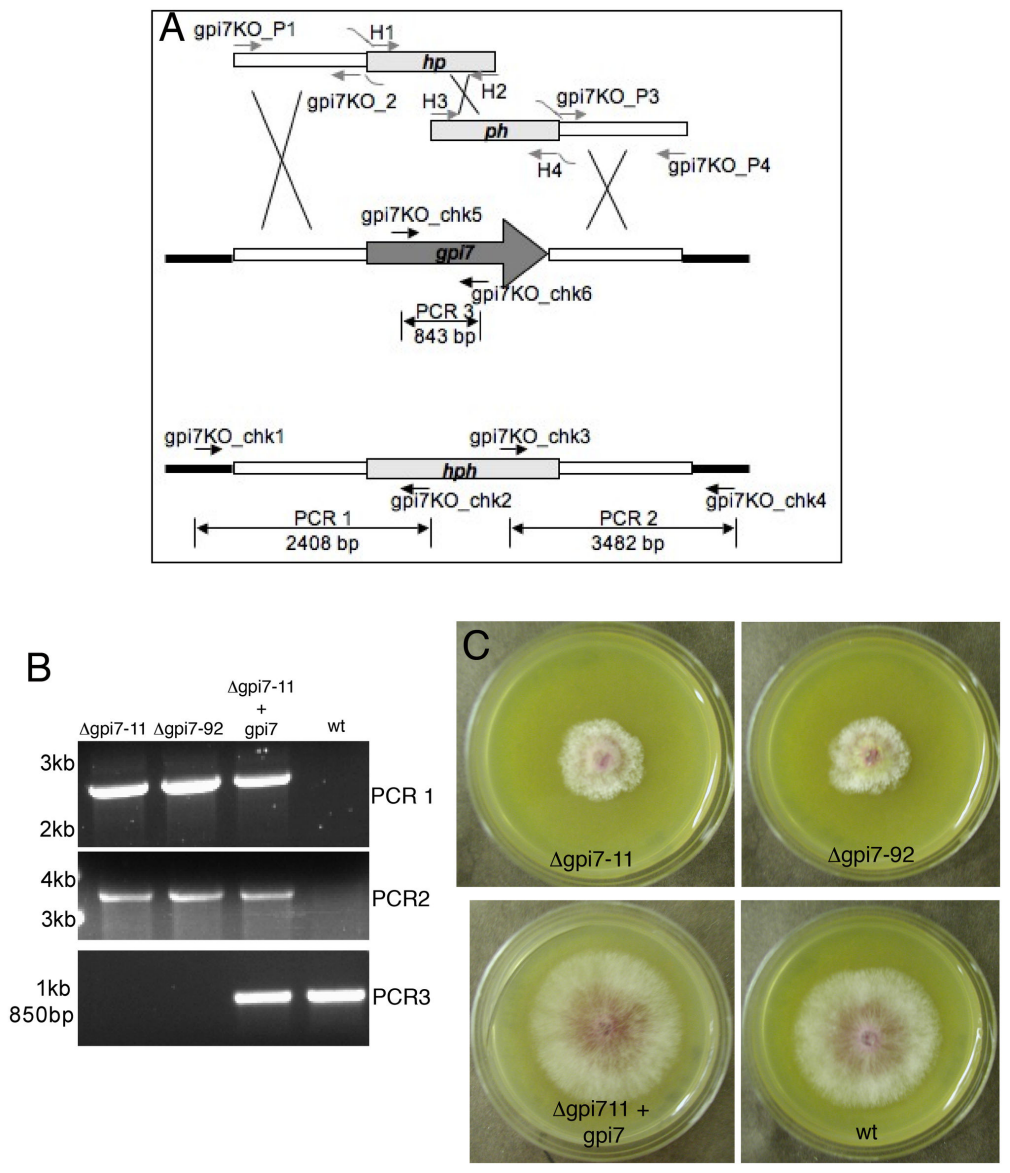
Replacement of *F. graminearum* *gpi7* with a hygromycin phosphotransferase (hph) marker. **A.** Gene replacement of *gpi7* using a split-marker approach. White bars represent genomic regions upstream and downstream of the *gpi7* coding sequence that were amplified and fused to segments of the hph cassette. Black bars represent genomic regions outside of the replacement construct. The deletion constructs were amplified with primers gpi7KO_P1-gpi7KO_P4 and H1-H4. Confirmation of gene replacement was tested with primers gpi7KO_chk1-gpi7KO_chk6. **B.** Results of diagnostic PCRs performed with primers gpi7_chk1-gpi7_chk6 (PCR reactions 1-3; see panel A). ‘gpi7-11’ and ‘gpi7-92’ are two independent Δ*gpi7* mutants. ‘gpi7-11+gpi7’ is strain gpi7-11 complemented with a complete *gpi7* coding sequence. ‘wt’ refers to strain PH-1. Numbers on the left represent the migration of standard DNA markers. **C.** Colonial phenotypes of Δ*gpi7* mutants after 3 days of growth on V8 medium at room temperature.

The proteome of *F. graminearum* was searched for orthologues of the *S. cerevisiae* Gpi3 and Gpi7. The search yielded FGSG_00960.3 (*gpi3*) and FGSG_02509.3 (*gpi7*). Primers were designed to replace these genes with a hygromycin phosphotransferase (hph) marker using a split-marker approach described previously [27] (Figure 1). Proper incorporation of the hph cassette at the *gpi7* locus was assessed using primers gpi7KO_chk1-gpi7KO_chk6 and Taq polymerase (Invitrogen) according to the manufacture’s instructions (Figure 1; Table S1). The Δ*gpi7* strain gpi7-11 was complemented with plasmid pBR31.1, which was generated by ligating a full-length copy of *gpi7* including 1kb of upstream and downstream regulatory sequences into the *Cla*I site of plasmid NatXho1-1 containing a nourseothricin resistance marker. The complementing strain possesses an ectopic integration of this plasmid.

For transmission electron microscopy, macroconidia were pelleted and fixed for 24 hours in 2.5% gluteraldehyde overnight at 4°C prior to processing. The samples were then fixed for one hour in 1% OsO_4_ at room temperature. Samples were subsequently washed with water and serially dehydrated with ethanol (25%-100%) and dried with CO_2_. The samples were embedded in LR-white and polymerized for three days. Ultrathin sections were cut and embedded on 200-mesh copper grids. Samples were stained with uranyl acetate and lead citrate and observed with a Hitachi H7500 microscope.

Sensitivity of Δ*gpi7* mutants and control strains to various concentrations of Glucanex (Sigma) was tested as follows. A stock concentration of 20 mg/ml Glucanex was made in sterilized distilled water, filter sterilized, and used to set working concentrations in YMA media. Macroconidia were then incubated at 28°C for 10 hours in YMA with different Glucanex concentrations, and hyphal growth patterns were observed microscopically. 

### Pathogenicity assays on wheat plants

Wheat plants (variety ‘Norm’) were grown under standard greenhouse conditions until anthesis (~7 weeks). At anthesis, the third full spikelet from the base of the head was inoculated at the two outer florets with 10 μl of a 1 X 10^5^ macroconidial suspension in 0.01% Tween 20. Inoculated heads were then covered with a plastic bag to create a humid environment; bags were removed after three days. Two weeks after the bags were removed, heads were removed and symptomatic spikelets (chlorotic and/or scabbed) were counted as a percentage of the total number of spikelets on the head. Two experiments were performed with 8 heads inoculated per strain tested. Spikelets were removed from the rachis, and the inoculated node +/- one node was cut and processed for light microscopy. The rachis sections were cleared/fixed for two days in a 3:1 ethanol:acetic acid solution in a 24-well cell culture dish at 75 RPM at room temperature. The sections were further fixed for two days in a 5:1:1 ethanol:acetic acid:glycerol solution under the same conditions. Thin sections were then sliced from the rachis with a razor blade and the aid of a dissecting scope. The thin sections were stained overnight with a lactophenol blue solution (33% lactic acid, 33% phenol, 0.01% trypan blue) under the conditions described above. The thin sections were then washed for four hours in 60% glycerol and then mounted in 60% glycerol and observed with an Olympus BX51 microscope under bright field conditions. 

Saprophytic growth of mutants was tested on wheat heads that were removed from plants and frozen. Frozen heads were allowed to thaw at room temperature, sanitized with UV irradiation for 30 seconds, and inoculated as described above. Heads were placed in 150 mm diameter Petri dishes with a moist piece of filter paper, covered with parafilm, and incubated at 24°C in a 12:12 photoperiod for five days. Four heads were inoculated for each strain tested. Rachis samples from these wheat heads were processed for microscopy as described above. Infection related morphogenesis was assessed *in vitro* by inoculating previously frozen wheat glumes with macroconidial samples. Glumes were then cleared, fixed, and stained as described above. Samples were assessed for coral-like subcuticular hyphae and bulbous infection hyphae.

### Prediction of GPI-anchored proteins and internal repeats within the GPI-proteome

The amino acid sequences of all predicted *F. graminearum* proteins were downloaded (www.broad.mit.edu) and analyzed for the presence of a predicted C-terminal GPI-anchor addition signal [7]. To predict the function of identified candidates, BLAST searches (www.ncbi.com) were used to identify homologues in yeasts and other fungi. The presence of a predicted N-terminal signal peptide was assessed using SignalP 3.0. Internal repeats were predicted with E-tandem software (EMBOSS) with a cutoff score of 20. Primers 8844_5/8844_6 and 1588_F/1588_R were used to amplify repeat regions in FGSG_08844 and FGSG_01588 respectively (Table S1). Amplified regions were subsequently cloned into TOPO 2.1 (Invitrogen) and submitted to University of Nebraska-Lincoln Genomics Core Research Facility for sequencing. Sequences were analyzed and aligned using MacVector software (MacVector, Inc).

 The FGSG_08844, FGSG_01588, and FGSG_00576 genes were replaced with a hygromycin phosphotransferase marker using the split-marker strategy as described above for the *gpi3* and *gpi7* genes [27] (Figure 1). The primers used to generate the necessary constructs and confirm gene knockouts are listed in Table S1. 

## Results

### Global disruption of GPI-anchoring in *F. graminearum*

In order to test the significance of GPI-APs on a global scale, genes encoding members of the GPI processing complexes were considered for characterization. Several proteins are responsible for constructing and ornamenting the GPI-anchor precursor and target protein during the maturation of GPI-APs [31]. Not surprisingly, several of these gene products are essential for viability in yeast and filamentous fungi. However, a few have been shown to result in viable deletion mutants, namely, *gpi3* and *gpi7* [20,32]. Accordingly, deletion of these two genes was attempted in *F. graminearum*. The orthologous *Saccharomyces cerevisiae* proteins were used to find the Gpi3 (FGSG_00960) and Gpi7 (FGSG_02509) sequences in *F. graminearum* (http://www.broadinstitute.org/annotation/genome/fusarium_group/MultiHome.html). FGSG_00960 is a predicted 501 amino acid protein that possesses 53% identity and 69% similarity to yeast Gpi3, with a BLAST e-value of e^-145^. FGSG_02509 is a predicted 783 amino acid protein that possesses 27% identity and 45% similarity to yeast Gpi7 with a BLAST e-value of e^-64^. Like Gpi7, FGSG_02509 also contains 11 predicted transmembrane domains (TMHMM predictor; data not shown) and a predicted signal peptide (SignalP 3.0; data not shown). Hereafter, we refer to FGSG_00960 and FGSG_02509 as *gpi3* and *gpi7*, respectively. Two viable and independent Δ*gpi7* mutants (gpi7-11 and gpi7-92) were recovered after transformation and isolated from single germinating macroconidia on hygromycin containing media (Figure 1), whereas no viable Δ*gpi3* mutants could be recovered. All three of the hygromycin-resistant *gpi3* transformants contained the *gpi3* coding sequence, which could not be eliminated through single macroconidial isolations (data not shown). This suggests that *gpi3* may be an essential gene in *F. graminearum*.

**Figure 2:**
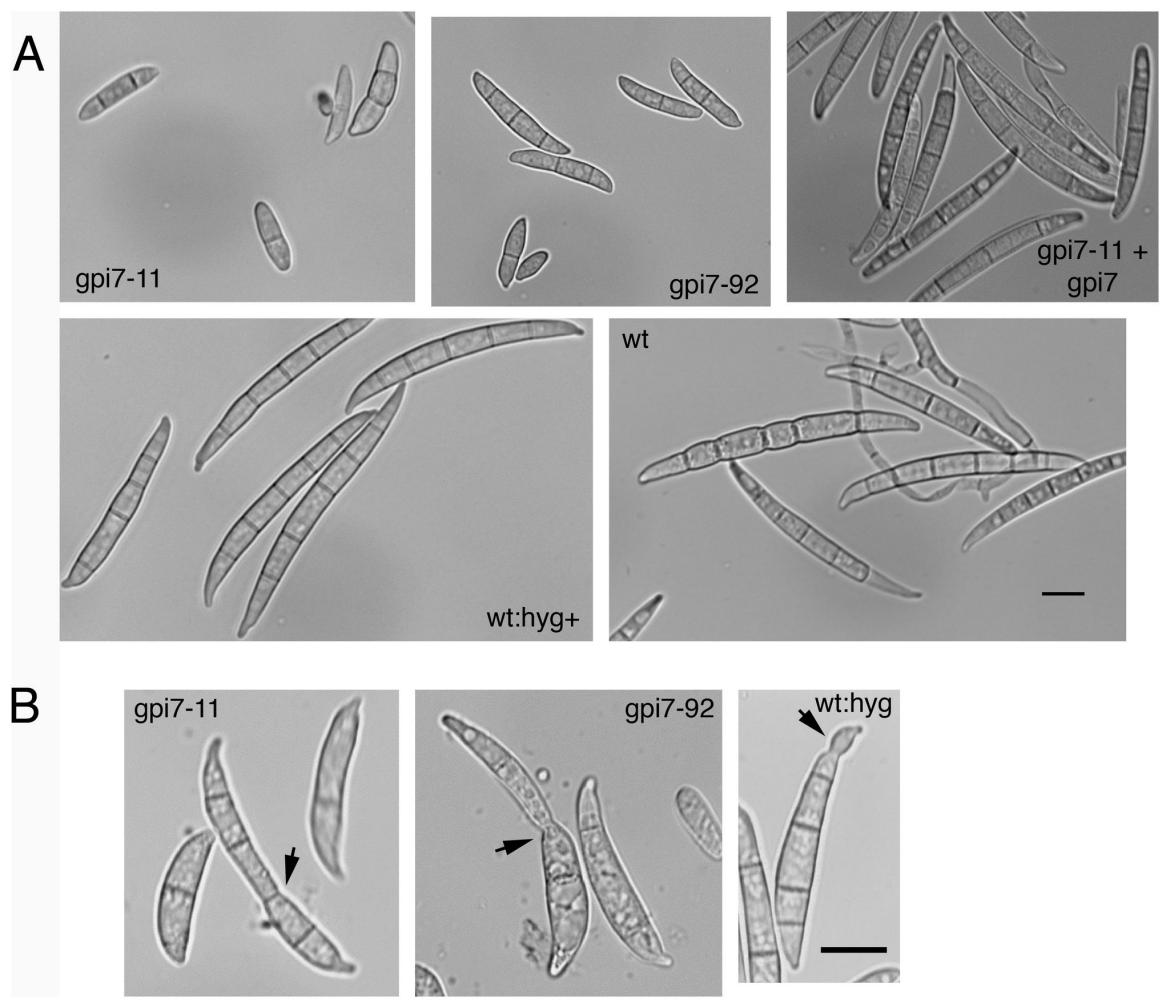
Macroconidia abnormalities of Δ*gpi7* mutants. **A.** Reduced length of macroconidia produced by Δ*gpi7* mutants gpi7-11 and gpi7-92. All micrographs in panel **A** are the same scale. **B.** Cell separation defect of Δ*gpi7* macroconidia. Some macroconidia appeared to be two macroconidia ‘fused’ together (black arrows). Roughly 10% (see Table 2) of the macroconidia from both gpi7-11 and gpi7-92 displayed macroconidia of this phenotype. Note that the macroconidia scored ‘aberrant’ for wt:hyg+ strain P2 were restricted to small protrusions at the macroconidial tip, whereas the phenotypes seen in the Δ*gpi7* mutants were more drastic. All micrographs in panel **B** are at the same scale. Scale bars = 10 μm.

**Figure 3:**
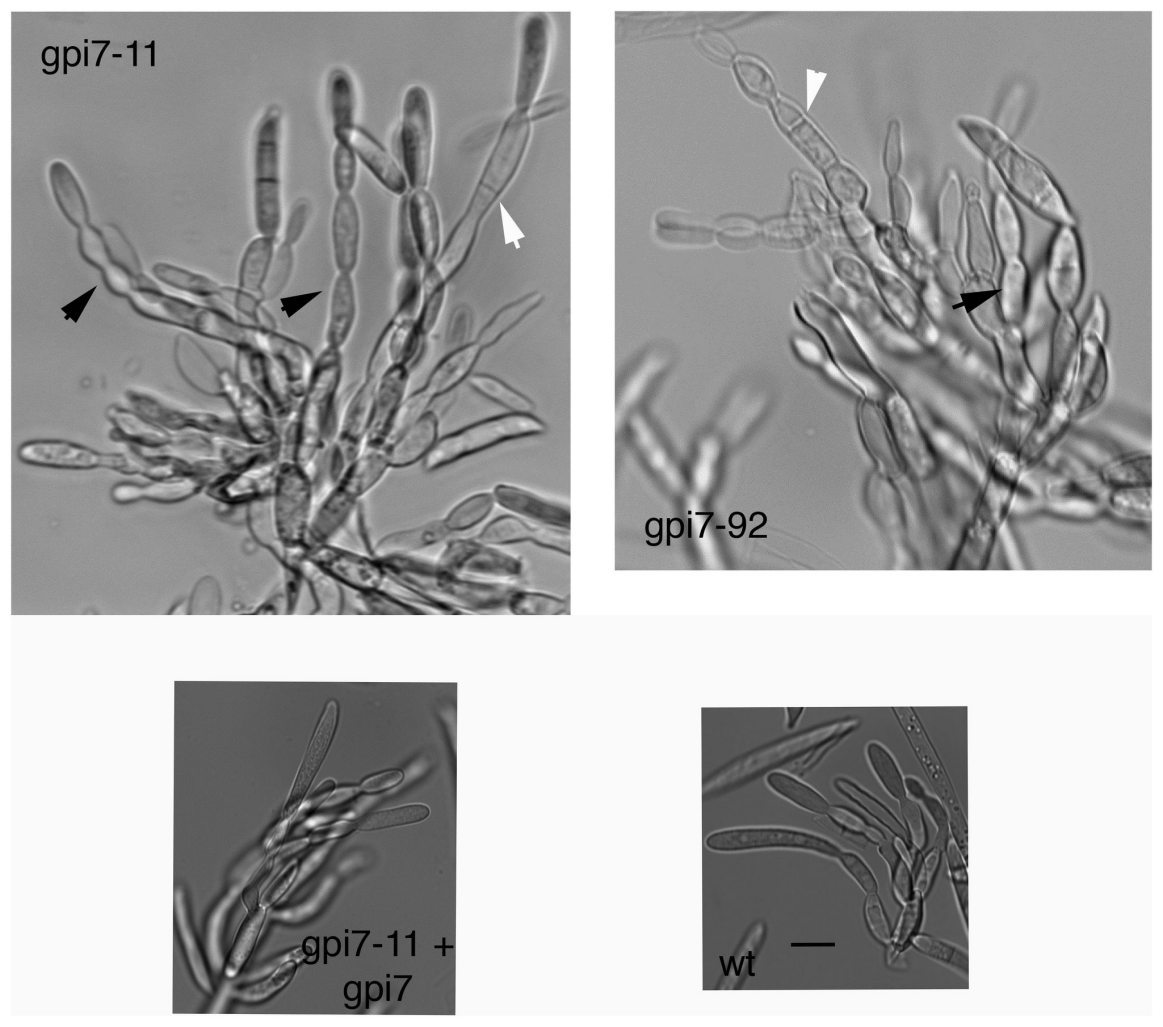
Conidiophores of Δ*gpi7* mutants. Note the long chains produced by Δ*gpi7* strains gpi7-11 and gpi7-92 (black arrows), compared to the short chains produced by the complemented strain (gpi7-11 + gpi7) and wildtype strain (wt). Also, conidiophores of the Δ*gpi7* mutants displayed septation within the conidiophores (white arrows), while such septation sites are not typical within the conidiophores of wildtype strains. All panels in same scale. Scale bar = 10 μm.

 The Δ*gpi7* mutants displayed a few striking phenotypes. First, the radial growth rate was slower than that of the wildtype and the complemented mutant (Figure 1C; Table 1). However, biomass production, as well as rates of conidial germination and hyphal growth, were similar to control strains when grown in liquid media (Table 1 and data not shown). There was no difference in the amount of perithecia or macroconidia produced by the Δ*gpi7* strains, though the macroconidia that were produced by gpi7-11 and gpi7-92 displayed an aberrant morphology compared to the slender macroconidia of wildtype and complemented strains (Table 1; Figure 2A). Furthermore, ~9% macroconidia from strains gpi7-11 and gpi7-92 displayed a “fused” phenotype, that is to say, it appeared that they were connected to another macroconidium (Figure 2B; Table 1). This phenotype may represent a macroconidium budding defect in which Δ*gpi7* mutants fail to completely separate from the phialides of conidiophores. Indeed, the conidiophores produced by Δ*gpi7* strains displayed long chains of phialides and aberrant septation locations (Figure 3). 

**Table 1:** Phenotypic data for Δ*gpi7* mutants.

**Strain** ^1^	**Dry Biomass** ^2^ **, mg (SD**)	**Colony Diameter** ^3^ **, cm (SD)**	**Macroconidia Concentration** ^4^ **(SE**)	**Macroconidia Length** ^5^ **, μm (SD**)	**% Abberrant Macroconidia** ^6^ **(SE**)	**Perithecia (SE**)^7^
*gpi7*-*11*	156.7 (20.2)	1.9 (0.1)	1.13x10^5^ (14.2x10^3^)	19.2 (5.5)	10.1 (1.2)	2.7 (0.2)
*gpi7*-*92*	131.7 (44.4)	1.9 (0.1)	1.22x10^5^ (6.4x10^3^)	24.1 (7.1)	8.4 (0.7)	2.8 (0.3)
*gpi7*-*11* + *gpi7*	ND	3.4 (0.06)	0.96x10^5^ (4.4x10^3^)	47.2 (8.9)	1.2 (0.1)	ND
wt:hyg	ND	ND	ND	54.4 (8.5)	1.9 (0.3)	2.8 (0.1)
wt	136.7 (12.7)	3.3 (0.12)	0.91x10^5^ (8.1x10^3^)	49.1 (11.0)	1.8 (0.4)	3.1 (0.3)

1 – ‘gpi7-11’ and ‘gpi7-92’ are Δ*gpi7* strains; ‘gpi7-11 + *gpi7*’ is strain gpi7-11 complemented with a full length *gpi7* coding sequence. ‘wt’ is strain PH-1. ‘wt:hyg’ is wt strain PH-1 expressing a hygromycin phosphotransferase cassette.

2 – Mean dry biomass recorded after 3 days growth in liquid YMA media. Three replicates were analyzed for each strain. SD = standard deviation.

3 – Mean colony diameter of strains after 3 days growth on solid V8 medium. Three replicates were analyzed for each strain.

4 – Mean macroconida concentration of liquid CMC cultures growing at room temperature after 5 days. Three replicates were analyzed for each strain. SE = standard error.

5 – Mean macroconidia length of strains grown in liquid CMC. A minimum of 50 macroconidia were measured for each strain.

6 – Mean percentage of macroconidia displaying a “fused” phenotype (see Fig. 5.3). Over 1000 macroconidia were analyzed over 3 replicates for each strain.

7 – Mean density of perithecia on carrot agar plates (no. perithecia mm^-1^). Three replicates were analyzed for each strain.

ND = not determined.

**Table 2:** Number of predicted GPI-anchored proteins in the proteome of *F*. ***graminearum***.

	**Signal Pep** **	**No Signal Peptide** **	**Total**
**Predicted orthology** *	57	24	81
**No predicted orthology** *	90	34	124
**Total**	147	58	205

* - Predicted orthology or no predicted orthology to proteins in NCBI database

** - Predicted signal peptide when tested with SignalP 3.0.

**Figure 4:**
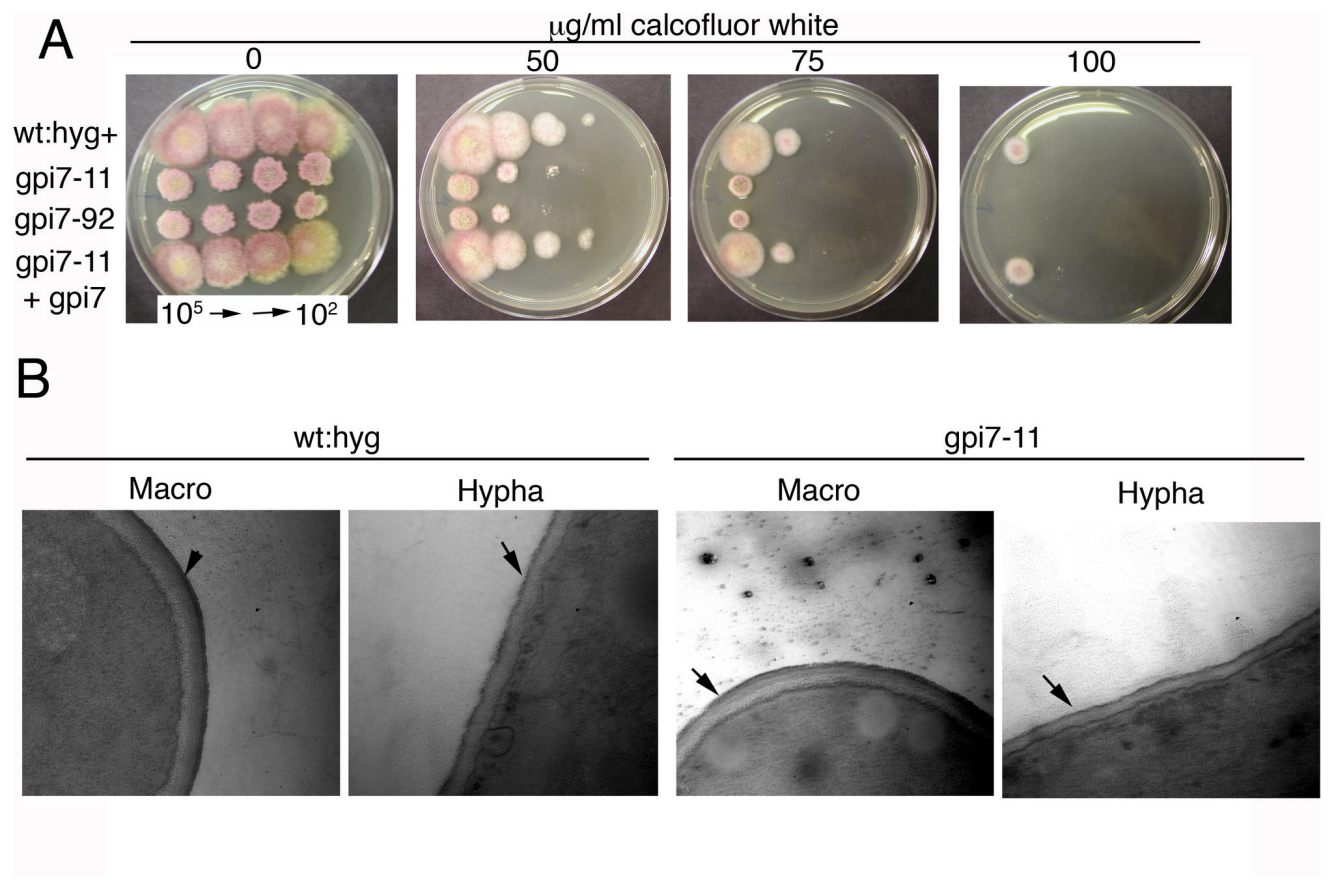
Cell wall defects of Δ*gpi7* mutants. **A.** Increased sensitivity of Δ*gpi7* mutants gpi7-11 and gpi7-92 to the cell wall disturbing agent calcofluor white compared to the complemented strain (gpi7-11 + gpi7) and hygromycin-resistant PH-1 strain (wt:hyg+). 7 μl of macroconidial suspensions of different concentration were serially spotted onto media. **B.** Transmission electron micrographs of cell walls in wildtype and Δ*gpi7* mutant macroconidia and hyphae. Black arrows point to the outer protein layer of the cell wall. There were no gross morphological changes observed in the cell wall of the Δ*gpi7* mutant. Scale bar = 500 nm.

 In order to test the Δ*gpi7* mutants for cell wall defects, their sensitivity to the cell wall disruption agents calcofluor white and Congo red was assessed. Both Δ*gpi7* mutants displayed increased sensitivity to calcofluor white (Figure 4A), but not Congo red (data not shown). This phenotype was complemented when the *gpi7* coding sequence was reintroduced into the gpi7-11 strain, indicating that the sensitivity was due to the deletion of the *gpi7* gene (Figure 4A). Despite the sensitivity to calcofluor, no defects were obvious in the cell wall when observed by staining growing hyphae with calcofluor white (data not shown) or by transmission electron microscopy (Figure 4B). These data suggest that deletion of *gpi7* has a subtle effect on composition of the cell wall in *F. graminearum*. 

### Reduced virulence of Δ*gpi7* mutants

**Figure 5:**
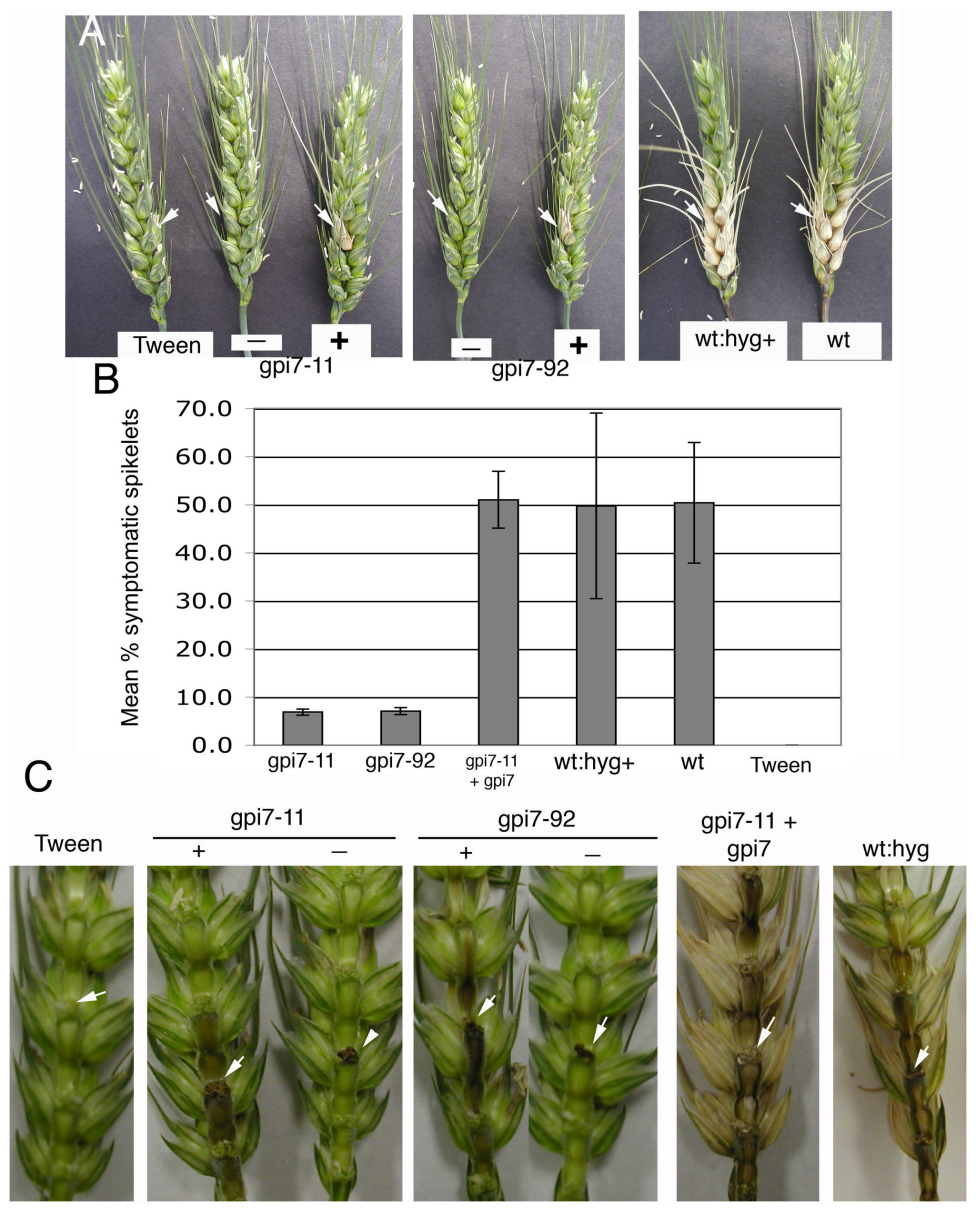
Virulence of Δ*gpi7* mutants gpi7-11 and gpi-92. **A.** Inoculated wheat heads two weeks after inoculation. Tween=mock inoculated negative control. Regarding Δ*gpi7* mutants, “-“ refers to heads exhibiting some black scabbing on the inoculated spikelet, but little to no chlorosis, while “+” refers to heads that displayed chlorosis at the inoculated spikelet. White arrows indicate inoculated spikelets. **B.** Mean percentage of symptomatic spikelets per inoculated head. Across two experiments, *n* = 13, 19, 8, 8, 19, 19 for gpi7-11-, gpi7-92-, gpi7-11+gpi7-, wt:hyg+ -, wt-, and tween-inoculated heads, respectively. Error bars = +/- standard deviation. **C.** Spread of symptoms through the rachis of wheat heads. Florets were removed from the inoculated side of the wheat head. Note that symptoms in those heads scored “-“ did not advance into the rachis and that in those scored “+”, the advancement was less than that of control strains. White arrows indicate nodes where inoculated florets were located.

Δ*gpi7* mutants were inoculated into the florets of wheat heads to test their virulence. Interestingly, heads inoculated with each Δ*gpi7* mutant resulted in two classes of symptoms, which we designated “+” and “-“. The “+” symptoms were characterized by complete chlorosis of the inoculated spikelet, whereas “-“ symptoms refers to some scabbing on the inoculated spikelet, but little to no chlorosis (Figure 5A). Across the two inoculation experiments, 46.6% and 63.2% were scored “-“ for gpi711 and gpi792 respectively, with the remainder scored “+”. Regardless of which class the inoculated wheat heads were assigned, chlorosis never advanced to florets beyond the inoculated node with Δ*gpi7* mutants, even three weeks after inoculation (Figure 5A; data not shown). This is in contrast to the control strains, which caused necrosis on several of the florets beyond the inoculated node (Figure 5A). Accordingly, the mean number of symptomatic spikelets was significantly lower than on heads inoculated with the complemented and wildtype strains (Figure 5B). These data suggest that the *in planta* growth of Δ*gpi7* mutants is hindered. 

Once *F. graminearum* invades florets, it advances to other florets via the rachis, or central “stem” that bears all the other florets [33]. Accordingly, we hypothesized that Δ*gpi7* mutants may be defective in accessing and/or proliferating in the wheat rachis. Wheat florets were removed to examine disease progression in the rachis. Interestingly, several of the wheat heads inoculated with Δ*gpi7* did not cause scabbing or chlorosis in the rachis (namely, those wheat heads that were scored “-“) (Figure 5C). In those wheat heads that displayed a chlorotic inoculated spikelet (i.e. scored “+”), scab symptoms did advance into the rachis, albeit not as prolifically as the in wheat rachises of heads inoculated with the complemented and wildtype control strains (Figure 5C).

**Figure 6:**
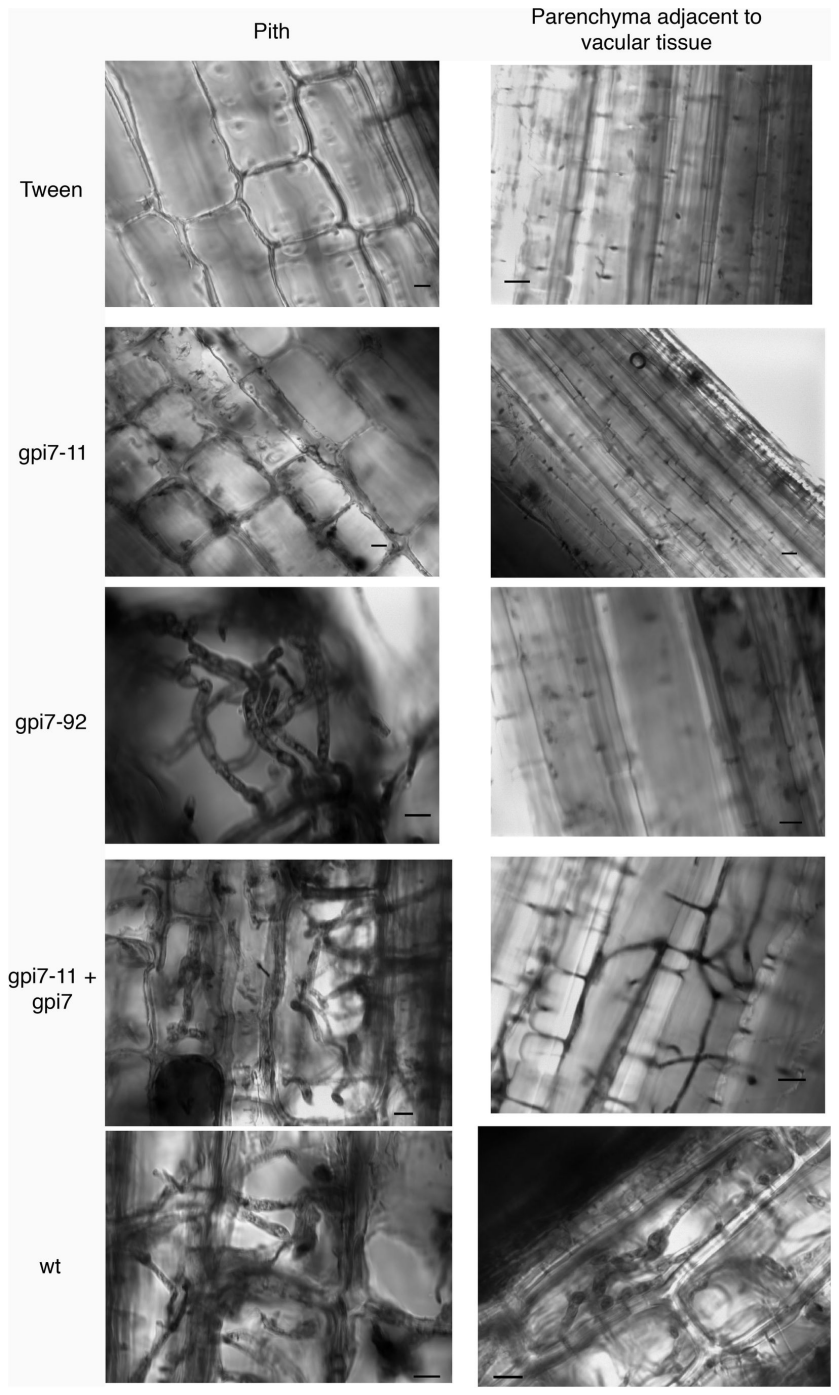
Hyphal invasion of rachis tissue. Rachis tissue adjacent to the inoculated spikelet was stained with lactophenol trypan blue and observed with bright-field microscopy. Both pith cells and parenchyma cell adjacent to vascular tissue were observed for the presence of hypha (note: vascular tissue stained with lactophenol blue, even in the mock-inoculated control, which made the observation of hyphae within vascular tissue difficult). ‘Tween’ represents mock-inoculated wheat heads; ‘gpi7-11’ and ‘gpi7-92’ were inoculated with these two Δ*gpi7* mutants; ‘gpi7-11 + gpi711’ were inoculated with the a complemented gpi7-11 strain; ‘wt’ were inoculated with strain PH-1.

Because the presence of symptoms does not necessarily correlate with the presence of the pathogen, the wheat rachises were examined microscopically for the presence of fungal hyphae. In those rachises scored “-“, hyphae were not observed in the pith tissue or the parenchyma cells adjacent to vascular tissue (seven heads of this class processed for microscopy; Figure 6, *gpi7*-*11*). In those wheat heads scored “+”, hyphae were readily seen in the pith tissue, but were not as prolific in the parenchyma cells adjacent to vascular tissue (six heads of this class processed for microscopy; Figure 6, *gpi7*-*92*). These data are in contrast to the control strains, in which hyphae readily invaded all tissues observed (four, four, and six heads observed for those inoculated by the complemented strain, wt:hyg+ strain, and PH-1 strain, respectively; Figure 6). These microscopy data, coupled with the symptoms described above, indicate that Δ*gpi7* mutants are hindered in breaching the floret-rachis barrier. When the mutants could breach this barrier, their spread within the rachis appeared limited to pith tissue.

**Figure 7:**
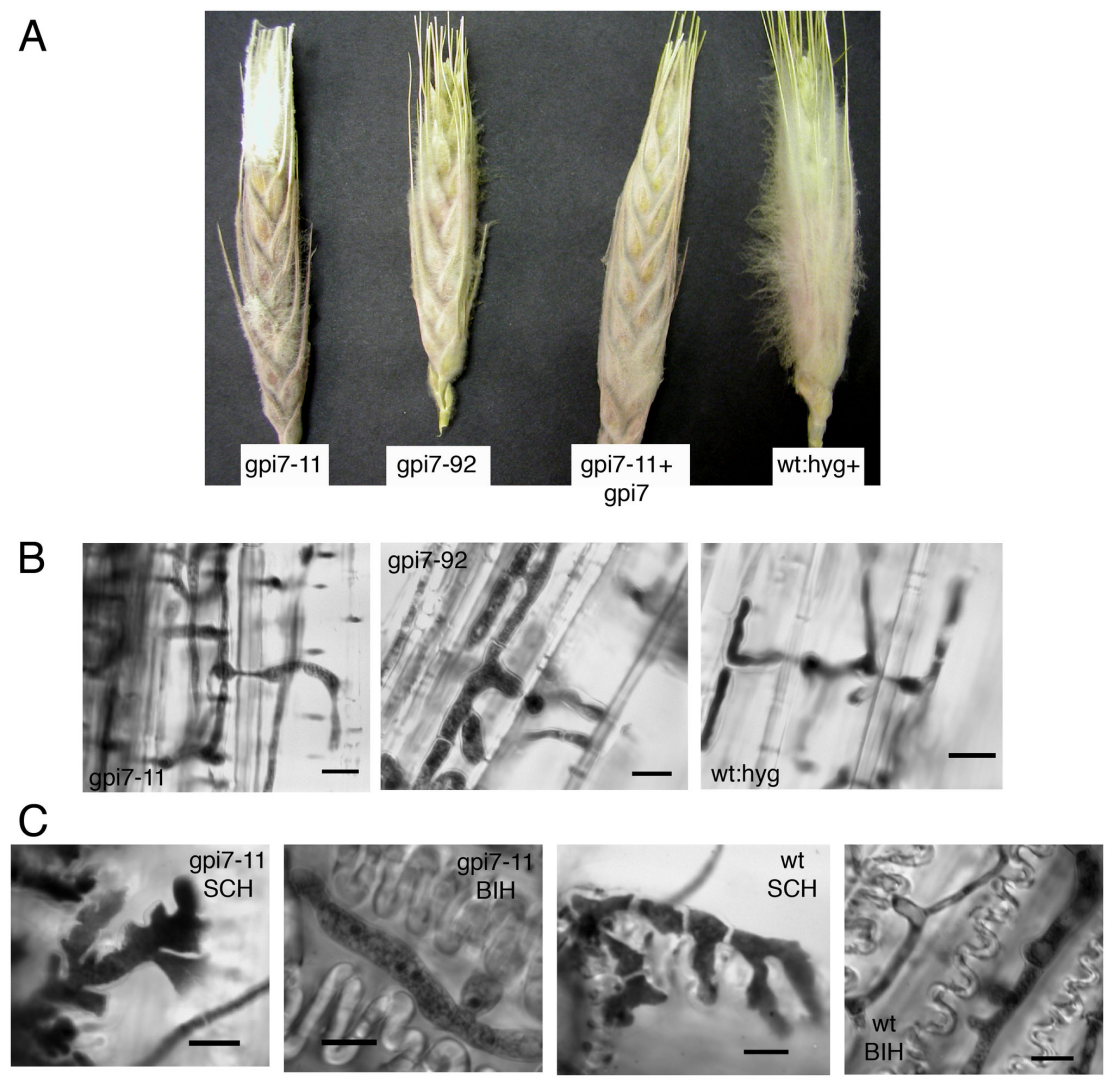
Saprophytic growth of Δ*gpi7* mutants and development of infection-related hyphae. **A**. Previously-frozen wheat heads that were inoculated at a single spikelet and observed 5 days post inoculation. Note the white aerial mycelia profilerating on wheat heads. **B**. Hyphal development within the rachis tissue of wheat heads depicted in panel **A**. Micrographs in panel **B** show the parenchyma cell adjacent to vascular tissue, which were not readily invaded by Δ*gpi7* mutants in living wheat plants. **C**. Development of infection-related hyphae on detached wheat glumes. SCH=subcuticular hyphae. BIH=bulbous infection hyphae. Scale bar = 10 μm in all micrographs.

It is possible that the reduced virulence of Δ*gpi7* mutants is caused solely by their slow growth rate. In order to compare the *in planta* growth of Δ*gpi7* mutants to saprophytic growth, previously frozen wheat heads were thawed and inoculated in the same manner as the wheat plants above. Hyphae of the Δ*gpi7* mutants spread prolifically throughout the wheat head, and readily invaded rachis tissues (Figure 7A, B). The Δ*gpi7* mutants were also capable of differentiating infection-related hyphae (subcuticular and bulbous) on detached wheat glumes (Figure 7C). Collectively, these data suggest that the virulence defect of Δ*gpi7* mutants is dependent upon living plant tissue and does not represent a general defect in infection-related development.

**Figure 8:**
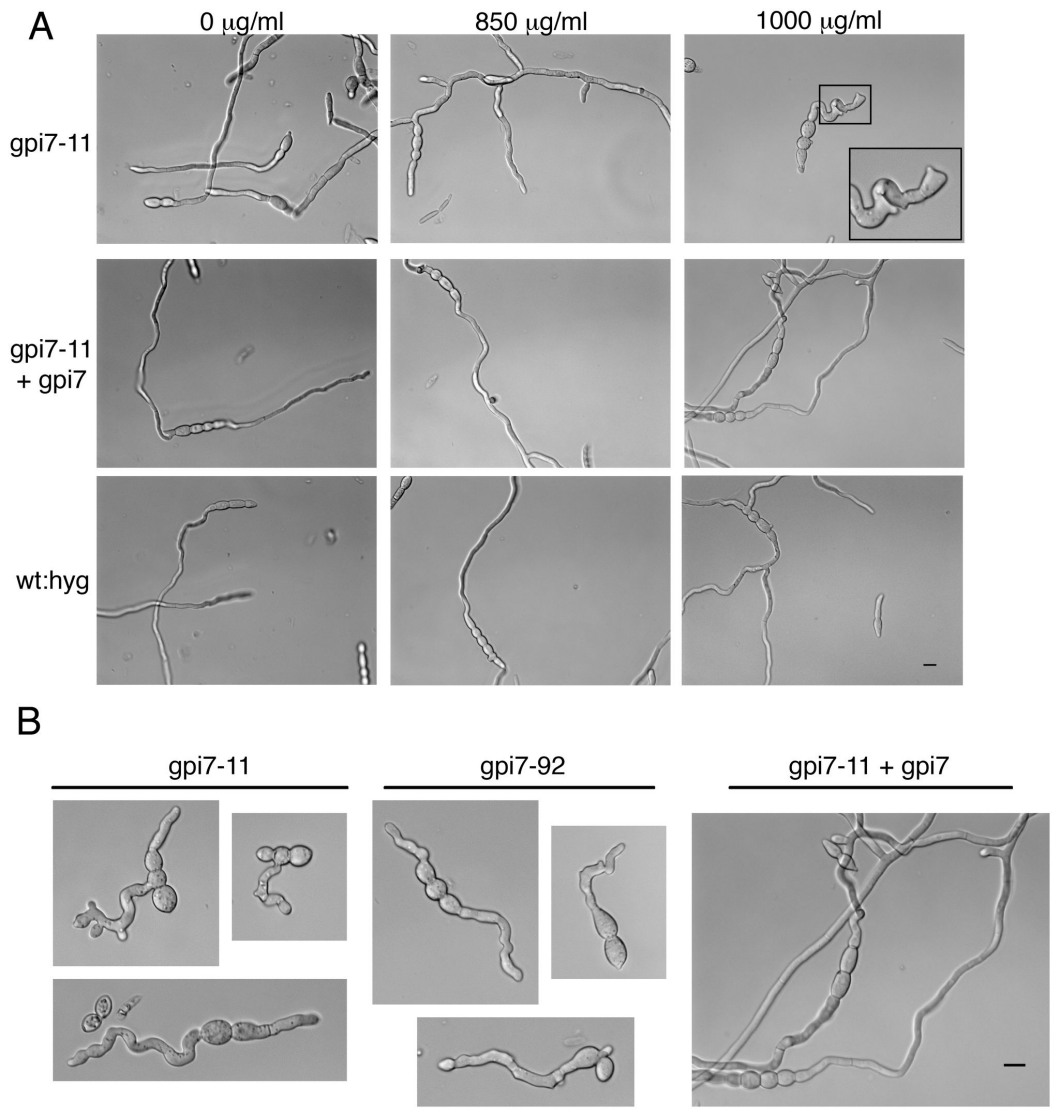
Hypersensitivity of Δ*gpi7* mutants to fungal cell wall degrading enzymes. **A**. Macroconidia were germinated in liquid media containing various concentrations of Glucanex. Images were captured 10 hour post inoculation. All micrographs in panel **A** are at the same scale. **B**. The hyphal phenotypes observed in Δ*gpi7* mutants at a Glucanex concentration of 1 mg/ml. All micrographs in panel **B** are at the same scale. Scale bars for panels A and B = 10 μm.

The inability of Δ*gpi7* mutants to proliferate *in planta* may be caused by an increased susceptibility of Δ*gpi7* mutants to plant defense compounds. Several genes encoding pathogenesis related (PR) proteins are up-regulated soon after inoculation with *F. graminearum*, including: peroxidase, chitinase, β-1,3 glucanase, and a thaumatin-like protein [34]. Δ*gpi7* mutants did not exhibit any differential susceptibility to oxidative stress caused by H_2_O_2_ or menadione (data not shown). However, the hyphal growth pattern of Δ*gpi7* mutants was drastically altered in the presence of Glucanex (Sigma; an enzyme cocktail containing both β-1,3 glucanase and chitinase). Whereas control strains were not affected by exposure to Glucanex, both germ tubes and hyphae of Δ*gpi7* mutants displayed aberrant morphologies (Figure 8).

### Prediction of GPI-anchored proteins in *F. graminearum*

Our characterization of Δ*gpi7* mutants highlights the importance of GPI-anchoring for growth, morphogenesis, and virulence in *F. graminearum*. Furthermore, these results suggest that one or more GPI-anchored proteins is important for virulence. To investigate this possibility, we first sought to obtain a global picture of the *F. graminearum* “GPI proteome” using an established bioinformatic approach [11,13]. Accordingly, all predicted *F. graminearum* proteins were screened as previously described for the presence of a possible GPI-anchor [7,16]. Of the ~14,000 proteins, 205 were predicted to have a GPI-anchor addition signal (1.5%; Table 2). Of these 205 proteins, 147 were also predicted to have a signal peptide, further supporting their cell-surface localization (Table 2).

Of the 147 predicted GPI-APs, 57 shared similarity to proteins of known function (Table S2). Similarly to GPI-AP inventories performed in other fungi, several of the proteins are predicted to be carbohydrate-modifying enzymes (Table S2). Several of the GPI-APs had functions that could conceivably be important for plant pathogenicity, including: cutinase, aspartyl proteases, rhamnogalacturonase, and proteins with the conserved cysteine-rich fungal extracellular membrane (CFEM) domain (Table S2)[35]. Considering that *F. graminearum* is (mostly) a necrotroph (i.e. must cope with oxidative stress of host cell death), we hypothesized that the lone predicted GPI-anchored superoxide-dismutase (SOD; FGSG_00576) might serve a crucial role in pathogenesis. However, when deleted, this gene failed to affect pathogenicity or sensitivity to the superoxide-generating agent menadione despite the observation that it was expressed in both the macroconidia and germinated spores of *F. graminearum* (Figure S1; data not shown). This suggests that either FGSG_00576 is not a true SOD, or it is functionally redundant with another SOD-encoding gene.

### Genes with internal repeats show variability among *F. graminearum* strains

The majority of predicted GPI-APs did not have a predicted function based on database similarity (Table 2). Notably, none of the predicted GPI-APs shared similarity to known fungal adhesins, whereas large families of GPI-anchored adhesins exist in *S. cerevisiae* and *C. albicans* [12]. In *S. cerevisiae*, the *flo* family of genes plays a significant role in adhesion, mating, and invasive growth [4]. As such, we predicted that a functionally analogous gene family might perform similar roles in *F. graminearum*, even though no orthologues exist based on primary sequence similarity. In order to assess which of the GPI-APs may possibly act in a *flo-*like function, they were queried for the presence of internal repeats. Such repeats are characteristic of fungal adhesions [13], and they are rich in Ser and Thr residues that function as glycosylation sites. Several GPI-APs were shown to contain repeats of different lengths (Table 3). 

**Table 3:** Top 10 (ordered by score) prediced GPI-APs also containing internal repeats.

**Accession #**	**Start** ^1^	**End** ^2^	**Score** ^3^	**Size of repeat** ^4^	**Number of repeats** ^5^	**Identity** ^6^
FGSG_06676	1086	1925	360	168	5	81.4
FGSG_01588	592	1119	216	48	11	75
FGSG_03359	215	790	196	192	3	83.7
FGSG_06479	662	961	191	75	4	94.3
FGSG_05232	1085	1588	163	63	8	72.4
FGSG_06952	1288	1662	156	75	5	80.8
FGSG_08844	824	1351	122	132	4	74.1
FGSG_06479	1825	2064	118	48	5	84.6
FGSG_00347	720	989	113	45	6	79.3
FGSG_01588	1156	1713	102	186	3	75.8

1 – nucleotide number (from A of ATG) where the repeat region starts

2 - nucleotide number (from A of ATG) where the repeat region ends

3 –Score of the repeats calculated by ETANDEM, which accounts for size, number, and identity of repeats.

4 – Number of nucleotides within a single repeat unit

5 – Number of repeat units

6 – Similarity between the repeat units

**Figure 9:**
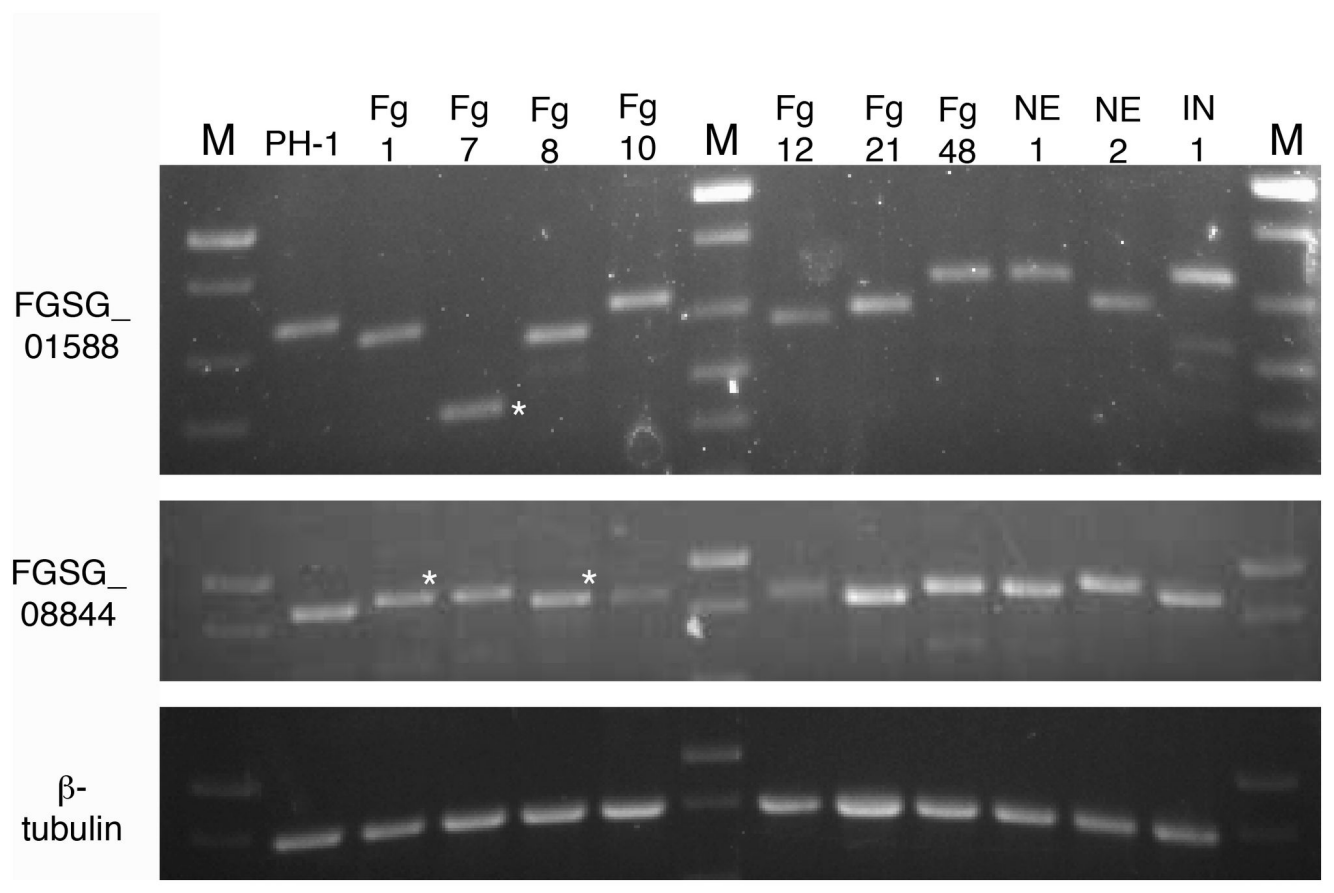
Intragenic variability in genes FGSG_01588 and FGSG_08844 among various *F. graminearum* strains collected in Nebraska (Fg1-Fg48, NE1-NE2, IN1). M=Invitrogen 1kb plus mass ladder. PH-1 = standard laboratory strain (genome sequenced). Those marked with an asterisk were cloned and sequenced (see Fig. 9).

**Figure 10:**
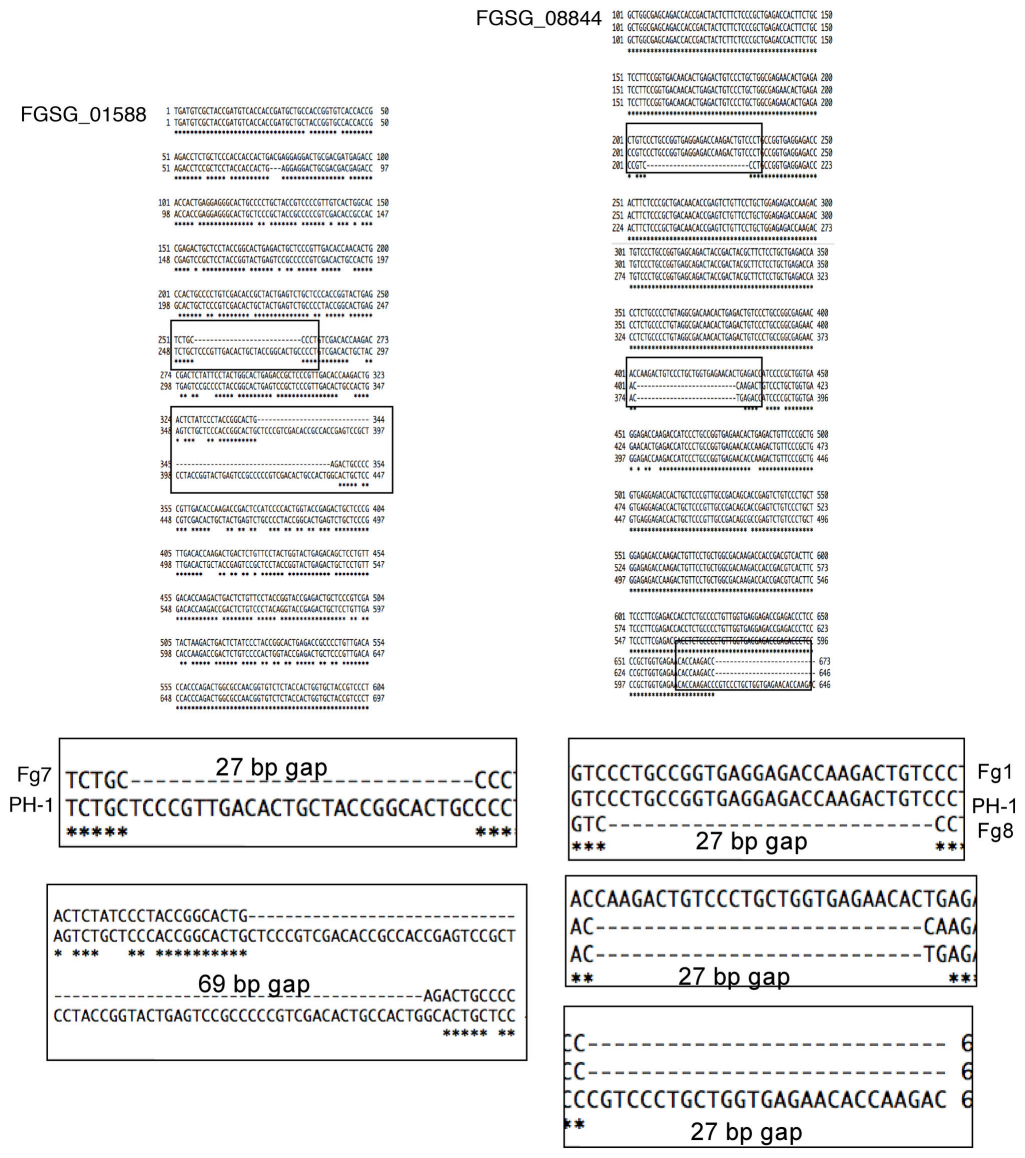
Differences in gene length of FGSG_01588 and FGSG_08844 are due to gaps within the variability region of the genes. Importantly, the gaps in DNA sequence occur in multiples of three, so that the rest of the coding region remains in-frame.

In order to determine which GPI-AP encoding genes of unknown function to characterize further, we tested the internal repeats for variability among *F. graminearum* strains. Variability in repeat length is a characteristic feature of GPI-anchored fungal adhesins [11,13,36]. Genes FGSG_01588 and FGSG_08844 contained differences in size among various *F. graminearum* strains (Figure 9), whereas the repeat region of FGSG_06676 did not display any detectable size differences (data not shown). For example, the amplified FGSG_01588 product from strain Fg7 was much shorter than that of strain PH-1 (Figure 9- asterisks). Likewise, the amplified FGSG_08844 product from strain Fg1 appeared slightly larger than wildtype. In order to determine the basis of the size differences, the amplified region of FGSG_01588 from strains PH-1 and Fg7 and the amplified region of FGSG_08844 from strains PH-1, Fg1, and Fg8 were cloned and sequenced. Sequencing data confirmed that the differences in band sizes are a result of gaps in sequence of the variability region. Importantly, these gaps occur in multiples of three, meaning that the rest of the protein sequence is still in frame (Figure 10). 

Based on their variability, FGSG_01588 and FGSG_08844 were selected for further characterization by gene deletion. First, we confirmed that these two sequences represented transcribed genes, as RT-PCR confirmed that they are both expressed in germinated macroconidia (data not shown). Although several deletion mutants were collected for both genes, no detectable phenotypes were observed when tested for: calcofluor white/Congo red sensitivity, SDS sensitivity, plant pathogenicity, infectious related development, sexual and asexual sporulation, or adhesion to polystyrene dishes (Figure S1). Also, FGSG_01588 and FGSG_08844 – GFP constructs failed to emit a clear localization signal (data not shown). Although expressed, these genes do not appear to be exclusively essential for the tested phenotypes. 

## Discussion

 The objective of this study was to investigate GPI-APs in the wheat pathogen *F. graminearum* and characterize their role in hyphal growth and virulence. To assess the general function of GPI-anchoring, we characterized the role of the PEA transferase Gpi7. Our data indicate that Gpi7 is required for proper cell wall organization, normal macroconidia formation, and full virulence on wheat heads. Consistent with what is known about these proteins in other fungi, we found that many of the predicted GPI-APs in *F. graminearum* encode carbohydrate-modifying enzymes that may contribute to hyphal growth by altering the carbohydrate skeleton of the cell wall. Also, several predicted GPI-APs have functions that may contribute to virulence, such as cutinase, CFEM domain proteins, and aspartyl proteases. We also demonstrated that at least two predicted GPI-APs (FGSG_01588 and FGSG_08844) contain internal repeat regions that display hypervariability among *F. graminearum* strains. 

In *S. cerevisiae* and *C. albicans*, *gpi7* is required for efficient covalent attachment of GPI-APs to the cell wall but is dispensable for the localization of membrane-bound GPI-APs [19]. These data suggest that the PEA group on the second mannose residue is critical for the covalent attachment of GPI-APs to the carbohydrate backbone of the fungal cell wall. Consistent with this cell wall defect, the *F. graminearum* Δ*gpi7* mutants generated in this study were more sensitive to the cell wall perturbing agent calcofluor white. Because calcofluor white exerts its effect by binding to chitin in fungal cell walls, we envision two possible reasons why Δ*gpi7* mutants are more susceptible to this reagent. First, they may have increased chitin content in their cell wall, as has been shown for other calcofluor-sensitive mutants [30]. The other possibility is that the outer protein layer of the cell wall in Δ*gpi7* mutants is more permeable to calcofluor white than cell walls of the wildtype strain. However, no obvious defects were seen in the outer protein layer of the cell wall when a Δ*gpi7* mutant was observed with transmission electron microscopy. Further investigation is necessary to identify the basis of the cell wall defect in Δ*gpi7* mutants of *F. graminearum.*

Another indication of a cell wall defect in the Δ*gpi7* mutant was the misshapen macroconidia and in some cases, their budding defect (i.e. ‘fused spores’ phenotype). This is similar to the budding/cell-separation defects observed in Δ*gpi7* mutants in *S. cerevisiae* and *C. albicans* [37,38]. The point at which conidia “bud” from the phialides likely requires significant modifications to the cell wall. Indeed, bud-site specific GPI-AP Egt2 is displaced in Δ*gpi7* mutants in *S. cerevisiae* [37]. The morphological deficiencies *of F. graminearum* Δ*gpi7* mutants described in this study support a similar role for cell wall proteins in macroconidial budding. Further investigation is necessary to determine which cell wall proteins contribute to this process. The inventory generated in this study supplies some likely candidates, such as chitinase (FGSG_10135), β1,3 glucanase (FGSG_02408), and β-glucosidase (FGSG_05085). All three enzymes have been implicated in yeast septation [39,40].

Very little is known about the role that cell wall proteins play in plant infection by *F. graminearum* and other plant pathogenic fungi. Our data suggest that proper cell surface organization in not absolutely required for pathogenicity, but is necessary for *in planta* proliferation of *F. graminearum*. We envision two possibilities as to the role that cell wall proteins play during plant invasion. First, it is possible that some cell wall proteins are enzymes that help digest components of plant cells. Consistent with this hypothesis, several putative GPI-APs are predicted to be cutinases, lysophospholipases, aspartyl proteases etc. A family of aspartyl proteases contributes significantly to the virulence of human pathogen *Candida glabrata*, which provides a precedent for their role in plant pathogenic fungi [24]. Despite their growth defect *in planta*, *F. graminearum* Δ*gpi7* mutants readily invaded dead host cells. This suggests that if wall-attached enzymes do contribute to virulence, they do so by targeting components of living cells. One class of candidates is the lysophospholipases (synonomous with phospholipase B; Table S2). These enzymes cleave the ester bonds on the acyl groups of phospholipids and contribute to the virulence of *C. albicans* and *C. neoformans* [21,41]. Their presence on the cell surface of *F. graminearum* may facilitate entry into host cells, as is the case with *C. albincans* lysophospholipase ca*PLB1* [41]. Genetic characterization of these candidates (aspartyl proteases and lysophospholipases) may prove difficult, as the presence of several representatives may permit functional compensation of any single deletion mutant. 

The second possible role of cell wall proteins may be as “barriers” against plant defense (e.g. PR) proteins. Indeed, several PR proteins encode enzymes that target components of the fungal cell wall (e.g. chitinases, β1,3 glucanses) [42]. The thaumatin-like proteins, are another class of PR proteins that includes osmotin, a defense protein produced by some plants in response to biotic and abiotic defenses [43]. Consistent with the “barrier” hypothesis of cell wall proteins, overexpression of a *S. cerevisiae* cell wall protein in the tomato wilt pathogen *F. oxysporum* increased its resistance to osmotin [25]. Thaumatin-like proteins have been shown to bind to β1,3 glucans, hence the outer shell of cell wall proteins may indeed act as a barrier to keep thaumatin-like proteins, β1,3 glucanases, and chitinases from reaching their targets. As such, a defect in cell wall anchoring of proteins may cause Δ*gpi7* mutants to be more susceptible to plant defense enzymes like β1,3 glucanase. Consistent with this notion, hyphal abnormalities occurred at lower Glucanex concentrations in Δ*gpi7* mutants compared to wildtype. Further investigation is necessary to determine which cell wall proteins are most abundant on the cell surface and what changes occur in the “cell wall proteome” during plant infection. 

Genes FGSG_01588 and FGSG_08844 contained internal repeat regions that varied among different *F. graminearum* strains isolated across Nebraska. Other studies have demonstrated similar hypervariability for select GPI-APs in yeasts *S. cerevisiae* and *C. albicans* [13,36] and the filamentous fungi *Aspergillus fumigatus* [11]. Although the function of such variability is unknown, it is predicted that it allows microorganisms to elude host defense responses [44]. Further functional studies and phylogenetic analyses of FGSG_01588 and FGSG_08844 should address this possibility, as well as revealing the potential utility of these variable repeat regions as diagnostic markers. 

Deletion of FGSG_01588 and FGSG_08844 failed to reveal any obvious phenotypes. This may be due to gene redundancy, as both genes cluster into families with other predicted GPI-APs, namely FGSG_03378 and FGSG_04824, respectively. These two genes were selected for investigation based on the fact that they contained characteristics of fungal adhesins (e.g. variable internal repeats, GPI-anchor, signal peptide) [12,44]. Members of the *S. cerevisiae flo* family share similar features and are involved in several cell-to-cell and cell-to-substrate interactions [4]. A similar approach was used to identify the gene Afu3g08990 in *A. fumigatus*, which contributed to the adhesion of conidiospores to the extracellular matrix of lung cells [11]. However, macroconidia of the ΔFGSG_01588 and ΔFGSG_08844 mutants also adhered just as tenaciously to polystyrene Petri dishes, which have been used as a model substrate for adhesion of spores of from several other plant pathogenic fungi [45-49]. The adhesive properties of macroconidia were compromised in the presence of surfactants SDS and Tween 20 (data not shown), suggesting that hydrophobicity plays a strong role in this adhesion process. Many fungal spores are coated in small proteins called hydrophobins that mediate their attachment to hydrophobic surfaces [50]. The function of hydrophobins has yet to be studied in *F. graminearum*, but it is possible that they contribute to the adhesion of macroconidia to hydrophobic surfaces. Other possible candidates discovered in this study are those with fasciclin domains (Table S2), which have been shown to mediate adhesion in the neuron cells of *Drosophila melanogaster* [51].

Collectively, our investigation highlights the importance of the GPI modification for the normal growth, development, and virulence of *F. graminearum*. However, at the same time, we failed to identify a single GPI-anchored protein that is essential for these processes.

## Supporting Information

Figure S1 **Phenotypic analysis of ΔFGSG_01588 and ΔFGSG_08844 mutants.** **A**. Responses to the fungal cell wall disturbing agent calcofluor white. 7 μl of macroconidial suspensions of different concentration were serially spotted onto media. **B**. Mean percentage of symptomatic spikelets per inoculated head. Error bars = +/- standard deviation.
(JPG)

Table S1 **Oligonucleotide primers used in this study.**
(DOC)

Table S2 **Predicted functions of GPI-anchored proteins.**
(DOCX)
