# Supplementary figures and images for: Glycosylphosphatidylinositol-Anchored Proteins in Fusarium graminearum: Inventory, Variability, and Virulence
Source: PLoS One. 2013 Nov 29;8(11):e81603. doi: 10.1371/journal.pone.0081603 (PMC3843709; doi:10.1371/journal.pone.0081603)

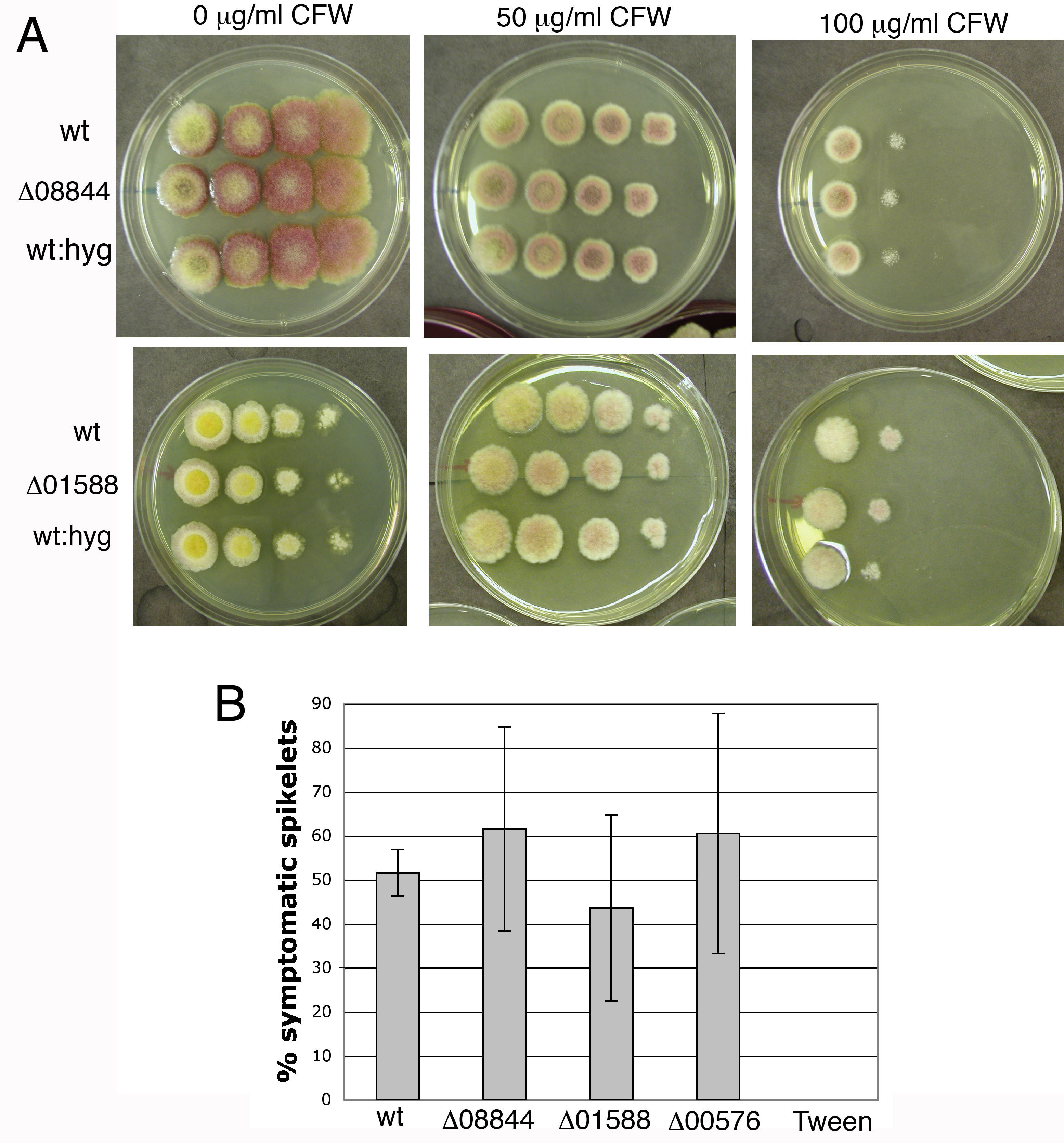

Supplement: Figure S1 — Phenotypic analysis of ΔFGSG_01588 and ΔFGSG_08844 mutants. A. Responses to the fungal cell wall disturbing agent calcofluor white. 7 μl of macroconidial suspensions of different concentration were serially spotted onto media. B. Mean percentage of symptomatic spikelets per inoculated head. Error bars = +/- standard deviation. (JPG) [file pone.0081603.s001.jpg]
