# Supplementary material for: Glycosylphosphatidylinositol-Anchored Proteins in Fusarium graminearum: Inventory, Variability, and Virulence
Source: PLoS One. 2013 Nov 29;8(11):e81603. doi: 10.1371/journal.pone.0081603 (PMC3843709; doi:10.1371/journal.pone.0081603)
Supplement: Table S1 — Oligonucleotide primers used in this study. (DOC) [file pone.0081603.s002.doc]

Supplemental Table 1. Primers used in this study.

| **Primer** | **Sequence (5' 🡪 3') ^1^** |
| --- | --- |
| gpi7KO_P1 | CAGGTTTGTTCTTCAAGCCGC |
| gpi7KO_P2 | AGTGTAAAGCCTGGGGTGCCTAATGAGTGA TTCACAGAGTCCACATCCATTGC |
| gpi7KO_P3 | TTTTACAACGTCGTGACTGGGAAAACCCTGTGTAAAGTTGGTTGCGGCTGG |
| gpi7KO_P4 | ATTCACGACTACTCCCTGCTCTGG |
| gpi7KO_chk1 | GTTGTTGGTGTATTTTGGACGGAG |
| gpi7KO_chk2 | AGGCGGCAGATGTGTTAGAAGC |
| gpi7KO_chk3 | CAAACTGTGATGGACGACACCG |
| gpi7KO_chk4 | TGATAGCGACGATGATGATACTGG |
| gpi7KO_chk5 | GCTTCCCTGTCTCCAAGAACAATC |
| gpi7KO_chk6 | TTTTGAGGCGAGCGAGTAGTCG |
| H1 | TCACTCATTAGGCACCCCAGG |
| H2 | GCTGCTCCATACAAGCCAACC |
| H3 | CGTTATGTTTATCGGCACTTTGC |
| H4 | CAGGGTTTTCCCAGTCACGAC |
| 8844_5 | TGACAAGCCCACTTTCCCTCAG |
| 8844_6 | AACGGTCTCGGTGACAACAACG |
| 1588_F | TGATGTCGCTACCGATGTCACC |
| 1588_R | ACAGTCTTGGTGGTGTAAGGGACG |
| Btub_F | TGCCTCCAGGGTTTCCAAATC |
| Btub_R | GCCTCAGTGAACTCCATCTCATCC |
| 08844KO_P1 | ATGCTGCCAGTGCTATCCTTCG |
| 08844KO_P2 | CCTGGGGTGCCTAATGAGTGAGTGAATGATTTGTTGGGGTTGG |
| 08844KO_P3 | GTCGTGACTGGGAAAACCCTGTTGGTGTGGACTTGGTGCTGAC |
| 08844KO_P4 | CAGAAAGCGGTTCAAATCACTCAC |
| 08844KO_chk1 | CGACACAGCAATCAACTACCCTATG |
| 08844KO_chk2 | TTCCCATTCCCATCGTGGTC |
| 08844KO_chk3 | GCAAAGGAATAGAGTAGATGCCGAC |
| 08844KO_chk4 | TTCAAGCAGCGAAAGGCGTC |
| 08844KO_chk5 | TGACAAGCCCACTTTCCCTCAG |
| 08844KO_chk6 | AACGGTCTCGGTGACAACAACG |
| 01588KO_P1 | CCTTTCTCTAATCTCCCCCTTGG |
| 01588KO_P2 | CCTGGGGTGCCTAATGAGTGATGTAATGAACGAATGGACTGCG |
| 01588KO_P3 | GTCGTGACTGGGAAAACCCTGTGTGACAACGCCCTGAACTCTG |
| 01588KO_P4 | TGTTTTACAGTGGGAGGAGCCC |
| 01588KO_chk1 | TCACCTCCTAACAAACTCCCCG |
| 01588KO_chk2 | TTCCACACAACATACGAGCCG |
| 01588KO_chk3 | GCCCTTCTGGATTGTGTTGGTC |
| 01588KO_chk4 | GCCGAAATAGGTGGATGTGTGAG |
| 01588KO_chk5 | TGAGGTCTACACCGTCACCAAGTG |
| 01588KO_chk6 | TGGCAAAGATACCAGCAGCG |
| 00576KO_P1 | GGTTTCGTTACGGGGATTGAAG |
| 00576KO_P2 | AGTGTAAAGCCTGGGGTGCCTAATGAGTGACGGCAAATGAGATGCTGAATG |
| 00576KO_P3 | TTTTACAACGTCGTGACTGGGAAAACCCTGAGATGTCGTGTTTCCTCGCCAG |
| 00576KO_P4 | CGTCAAAGCAATGATAGTCTCCTGC |
| 00576KO_chk1 | AAGCAAGTTGGACGGGATGAC |
| 00576KO_chk2 | AAAGCCTGGGGTGCCTAATG |
| 00576KO_chk3 | ATAGTGACTGGCGATGCTGTCG |
| 00576KO_chk4 | AGTTTTCCTTTTTGGGTCTGGC |
| 00576KO_chk5 | TGCCGAGAAGCCTGAATCTTG |
| 00576KO_chk6 | CACCTTGGTCTGCGAATCAATC |

^1^ – Underlined sequence represents ‘tails’ used to promote fusion of construct fragments to hygromycin phosphotransferase fragments.
