# Supplementary material for: Glycosylphosphatidylinositol-Anchored Proteins in Fusarium graminearum: Inventory, Variability, and Virulence
Source: PLoS One. 2013 Nov 29;8(11):e81603. doi: 10.1371/journal.pone.0081603 (PMC3843709; doi:10.1371/journal.pone.0081603)
Supplement: Table S2 — Predicted functions of GPI-anchored proteins. (DOCX) [file pone.0081603.s003.docx]

**Table S2.** Predicted functions of GPI-anchored proteins.

| **Predicted Domain/Function** | **# of hits** | **Accession #s** |
| --- | --- | --- |
| Appressorium-specific protein | 1 | FGSG_07861.3 |
| Aspartyl proteases | 5 | FGSG_04397.3 FGSG_03975.3 FGSG_01390.3 FGSG_11160.3 FGSG_05471.3 |
| β1,3 glucanase | 1 | FGSG_02408.3 |
| β-glucosidase | 1 | FGSG_05085.3 |
| Cacineurin-like phosphoesterase | 1 | FGSG_02069.3 |
| CFEM domain | 2 | FGSG_05175.3 FGSG_00588.3 |
| Chitinase | 1 | FGSG_10135.3 |
| Chitosanase domain | 1 | FGSG_04230.3 |
| Cu/Zn SOD | 1 | FGSG_00576.3 |
| Cutinase | 2 | FGSG_04077.3 FGSG_02666.3 |
| Cyclic phosphodiesterase | 1 | FGSG_02069.3 |
| Dak1 domain - kinase | 1 | FGSG_07017.3 |
| Exo-β-1,3-glucanase | 2 | FGSG_09366.3 FGSG_05292.3 |
| Fasciclin domain | 3 | FGSG_02125.3 FGSG_06592.3 FGSG_08535.3 |
| Gas1-like | 3 | FGSG_02022.3 FGSG_06037.3 FGSG_09980.3 |
| Glucanosyltransferase | 4 | FGSG_02720.3 FGSG_06873.3 FGSG_09289.3 FGSG_03017.3 |
| Glucose/sorbosone dehydrogenase | 1 | FGSG_09272.3 |
| Glycosyl hydrolase family 16 - O-glycosyl hydrolases | 1 | FGSG_05400.3 |
| Glycosyl hydrolase family 76 - α1,6mannase | 3 | FGSG_09648.3 FGSG_03714.3 FGSG_07016.3 |
| Glycosyl hydrolase family 81 - β1,3 glucanase | 1 | FGSG_09650.3 |
| GNAT homologue | 1 | FGSG_08757.3 |
| Herpes outer-envelope protein | 1 | FGSG_08844.3 |
| Infection structure specific protein | 1 | FGSG_05846.3 |
| Laminin domain, N-terminus | 1 | FGSG_1050.3 |
| Lysophospholipase | 2 | FGSG_02073.3 FGSG_10693.3 |
| Mid1 - Calcium ion channel protein | 1 | FGSG_07418.3 |
| Nucleoside transporter family | 1 | FGSG_12980.3 |
| Peptidase | 1 | FGSG_08454.3 |
| Pir-like | 1 | FGSG_02173.3 |
| Protease | 1 | FGSG_08245.3 |
| Rhamnogalacturonase | 1 | FGSG_00989.3 |
| SCP-like protein, involved in human and plant defense | 1 | FGSG_02744.3 |
| Sed1p homologue | 1 | FGSG_10772.3 |
| Solute Binding protein | 1 | FGSG_01588.3 |
| Uncharacterized conserved protein | 1 | FGSG_10759.3 |
| WSC-domain | 3 | FGSG_03884.3 FGSG_07784.3 FGSG_12938.3 |
| YeeE family; Bacterial membrane proteins of unknown function | 1 | FGSG_11292.3 |
| ZIP Zinc transporter | 1 | FGSG_02248.3 |
